# Moving towards a Comprehensive Approach for Health Literacy Interventions: The Development of a Health Literacy Intervention Model

**DOI:** 10.3390/ijerph15061268

**Published:** 2018-06-15

**Authors:** Bas Geboers, Sijmen A. Reijneveld, Jaap A. R. Koot, Andrea F. de Winter

**Affiliations:** Department of Health Sciences, University Medical Center Groningen, University of Groningen, P.O. Box 30.001, FA10, 9700 RB Groningen, The Netherlands; s.a.reijneveld@umcg.nl (S.A.R.); j.a.r.koot@umcg.nl (J.A.R.K.); a.f.de.winter@umcg.nl (A.F.d.W.)

**Keywords:** health literacy, model, intervention, review, health system, health professional, community, context, health outcomes

## Abstract

Low health literacy (HL) is associated with many negative health outcomes, and is a major challenge in public health and healthcare. Interventions to improve outcomes associated with HL are needed. In this paper, we aim to develop a comprehensive HL intervention model. We used a multimethod approach, consisting of (1) a literature review of articles listed in MEDLINE, presenting HL intervention models, (2) online consultation of international HL experts, and (3) two consensus meetings with members (*n* = 36 and 27) of a consortium studying HL among older adults (50+) in Europe. In our literature review, we identified twenty-two HL models, only a few of which focused explicitly on interventions. Sixty-eight health literacy experts took part in the online survey. The results from all three methods came together in a comprehensive HL intervention model. This model conceptualized interventions as potentially targeting five factors affecting HL outcomes: (1) individuals’ personal characteristics, (2) individuals’ social context, (3) communication between individuals and health professionals, (4) health professionals’ HL capacities, and (5) health systems. Our model is the first comprehensive HL model focused specifically on interventions. The model can support the further development of HL interventions to improve the health outcomes of people with low HL.

## 1. Introduction

Many adults worldwide have low levels of health literacy, meaning that they are less able to access, understand, appraise, and communicate information to engage with the demands of different health contexts to promote and maintain health across the life-course [1]. Large-scale survey studies have found prevalences of low health literacy of 36% in the United States [2], and between 29% and 62% in various European countries [3]. As low health literacy is associated with various negative health outcomes, including self-rated health [4], quality of life [5], and all-cause mortality [6], it is considered a major health challenge of the 21st century.

A growing consensus suggests that health literacy research should focus less on descriptive studies, and more on the development and testing of interventions. This requires a health literacy intervention model to conceptualize how interventions can achieve better health outcomes among people with low health literacy. Such a model may lead to a more comprehensive approach to improve health literacy outcomes and support intervention research. In the last two decades, many studies on the causes and consequences of low health literacy have been published, resulting in the development of various health literacy models [7,8,9]. However, these models focus mainly on identifying associations between health literacy and its determinants and outcomes. Models focused on identifying targets for interventions are scarce, and those available are of limited comprehensiveness.

**Figure 1:**
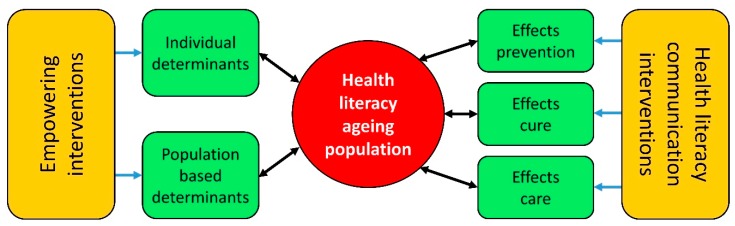
Provisional health literacy intervention model.

The authors conceptualized a provisional health literacy intervention model at the start of a European project entitled Intervention Research on Health Literacy among the Ageing Population (IROHLA; Figure 1). They developed this model based on their expertise and their experience with developing and implementing health promotion programs, as well as on existing literature on health literacy [3,8].

This provisional intervention model shows that health literacy levels are determined by both individual and population-based determinants, and by health systems. The model distinguishes between primary prevention (health promotion and protection), secondary prevention (detecting and treating health problems in an early stage), and tertiary prevention (softening the impact of existing health problems). The model includes two main types of interventions: (1) those aimed at empowering people by positively affecting individual or population-based determinants, and (2) those aimed at mitigating the negative consequences of low health literacy by improving the communication used in healthcare systems.

In this study, we aim to develop a comprehensive health literacy intervention model, using the provisional model as a starting point.

## 2. Materials and Methods

We used a multimethod approach to improve and build on the provisional health literacy intervention model. Three main methods were used:

1. A review of the literature on health literacy models.
2. Online consultation with health literacy experts.
3. Consensus meetings with members of the IROHLA consortium.

### 2.1. Setting

Part of the research activities for this study were conducted in the framework of the Intervention Research on Health Literacy among the Ageing Population (IROHLA) project. The IROHLA project (2012–2015) focused on improving health literacy for older adults above the age of 50 in Europe [10].

### 2.2. Literature Review

We conducted a search for articles presenting health literacy models by searching the MEDLINE electronic database from its inception through May 2013 (and updated in July 2017) for papers that included the term *health literacy* in their title and *framework*(*s*), *model*(*s*), *theory*, *theories*, *concept*(*s*), *pathway*(*s*) or *mechanism*(*s*) in their title or abstract. We defined a model as a graphical of textual representation of a concept, including its various aspects and/or its overlap or associations with other concepts.

The first author, BG, performed selection in three rounds: title screening, abstract screening, and full text screening. In the title screening round, BG was supported by research assistant JPMV. In case of doubts about inclusion, AFW acted as a second reviewer. This process was followed in the full text screening of five articles (7.1%). BG manually screened the reference lists of included articles for further articles meeting the inclusion criteria.

We included articles if they presented a model on health literacy: (1) based on evidence, a strong theoretical basis, or published literature, and (2) sufficiently generalizable to the broader adult population. We excluded articles if (1) the presented model was based on analyses of only a single dataset, (2) the model focused specifically on children or adolescents, (3) the type of health literacy addressed was insufficiently generalizable to health literacy in general, or (4) if no full text could be obtained. We imposed no restrictions with regard to article types (e.g., reviews, commentaries, empirical studies) or languages. However, other criteria led to the exclusion of all non-English articles before the full-text screening.

Data from the included articles were extracted by means of a form specifically developed for this review. Besides bibliographic information, the form captured the main aims and conclusions of the article, the theoretical basis for the model, the main focus and target group of the model, and its implications for interventions.

### 2.3. Online Consultation

We conducted an online survey among health literacy experts in the IROHLA consortium and among external health literacy experts. We identified the latter by searching the existing literature on health literacy and via recommendations by members of the IROHLA consortium.

The study encompassed two rounds consisting of closed and open-ended questions. We invited a total of 139 health literacy experts (34 members from the IROHLA consortium and 105 external experts) to participate in the first round (November 2012–February 2013). For the second round (May–August 2013), we invited 162 participants (60 from the IROHLA consortium and 102 external health literacy experts). The lower number of participants from the IROHLA consortium in the first round was because the formation of the consortium was still ongoing at that time.

In the first round, participants reflected on the concept of health literacy, and on its definition by Sørensen et al. (2012) [9]. Participants were also asked to list any constructs, theories, or existing best practices that they deemed relevant for developing health literacy interventions.

In the second round, participants again reflected on the concept of health literacy, but this time on its definition by Kwan (2006) [1]. Another definition of health literacy was chosen for the second round in order to get as much information as possible from the participants with regard to the elements they considered essential in a definition of health literacy. Participants were also asked to name the characteristics they considered most important in a health literacy intervention model and to reflect on an intermediate version of the model. Finally, the participants were shown a list of potentially modifiable determinants of health literacy, and asked to evaluate their relevance for health literacy interventions. The list of determinants was composed based on the results of the first round of the online survey.

### 2.4. Consensus Meetings

Consensus meetings were held in the Netherlands with members of the 21 organizations of the IROHLA consortium, in December 2012 in Groningen (*n* = 36), and in May 2013 in Amsterdam (*n* = 27). During these meetings, participants discussed intermediate versions of the health literacy intervention model. In an open discussion, all members present were invited to reflect on the model and provide suggestions for further improvements. Both consensus meetings took approximately one hour.

### 2.5. Analyses and Reporting

For each of the three main methods, we first describe the characteristics of the included participants. Next, per method, we report the findings regarding models on interventions aiming to improve the health outcomes of people with low health literacy. Finally, we amalgamate the findings of these three methods into the development of a final model.

## 3. Results

### 3.1. Literature Review

**Figure 2:**
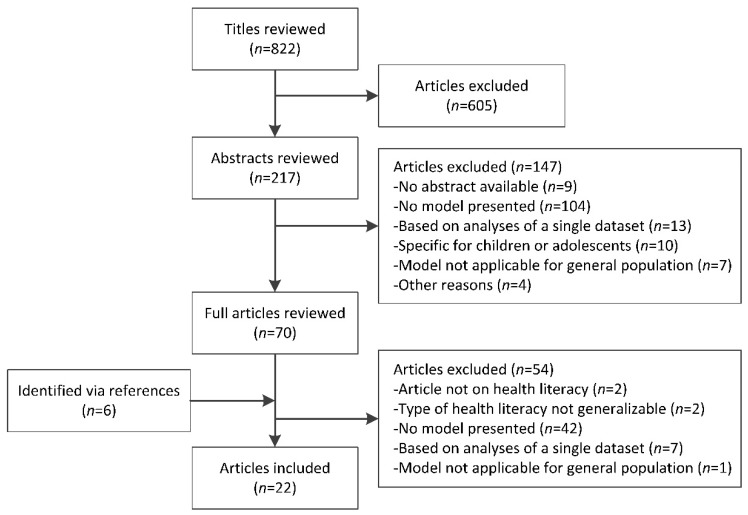
Flowchart of selection procedure.

In our search, we identified a total of 822 articles, 16 of which were included after three rounds of screening. Reference mining led to the inclusion of six additional articles, resulting in a total of 22 articles. The full selection process is presented in Figure 2.

The overall results of the literature review are shown in Table S1 in Supplemental Materials. For every article included in the review, this table contains the basis of the model as presented, a description of that model, and its implications for interventions, as stated by its authors.

Of the 22 models included in the literature review, most were based on existing literature on health literacy [7,9,11,12,13,14,15,16,17,18,19,20,21]. Others were primarily based on more general theories and concepts [8,22,23,24,25], concept mapping [26], practical experiences [27], or combinations of these sources [28,29].

Regarding the characteristics of the models, 16 primarily focused on associations of health literacy with determinants and outcomes [7,8,9,11,12,13,14,15,16,17,18,19,20,21,27,29], and of these, 15 included determinants of health literacy at the level of the individual (e.g., educational level, age, cognitive skills) [7,8,9,11,12,13,14,16,17,18,19,20,21,27,29]. Most of these models also included intermediate outcomes between health literacy and health outcomes (e.g., knowledge, health behaviors, self-care) [7,8,9,11,12,13,16,18,19,20,21,27,29]. Fifteen models included the role of health professionals and the health system [7,8,11,12,13,14,15,16,18,20,23,25,26,28,29]; this role was also the prime focus of four of these models [16,23,25,28]. One model specifically defined health literacy as the bridge between individual skills and abilities and the health system [15].

Most existing health literacy models do not focus explicitly on interventions. Many articles only briefly mention that their models are useful for the development of interventions, and only a few more specifically describe their models’ implications for interventions [8,24,27,28]. Von Wagner et al. (2009) describe how their model can be used in three domains of health actions (i.e., access and use of health services, patient–provider interactions, and management of health and illness), by giving examples of interventions in these three domains [8]. McCormack et al. (2017) present five strategies to intervene in health literacy, based on the most important constructs of their model (i.e., health literacy, patient engagement, socioecological levels) [24]. Rootman and Ronson (2005) describe several specific intervention targets and strategies (i.e., health communication, education/training, community and organizational development, and policy development) [27]. Finally, Vellar, Mastroianni, and Lambert describe the design and development of their framework for implementation specifically in the context of the Illawarra Shoalhaven Local Health District in Australia, and the results of this implementation [28].

### 3.2. Anonymous Online Consultation

A total of 39 health literacy experts participated in the first round of the online survey (response rate: 29%). In the second round, we received responses from 49 health literacy experts (response rate: 30%). Of the 68 respondents who participated in at least one round of the survey, the largest group (63%, *n* = 43) were academia. Other participants (37%; *n* = 25) were healthcare professionals and policy makers. Respondents resided in Europe (*n* = 48; 71%), the United States (*n* = 10; 15%), Canada (*n* = 5; 7%), and other countries (*n* = 5; 7%). Results revealed a considerable disagreement between respondents regarding the nature of health literacy. Some experts perceived health literacy as the skills and abilities of individuals to access, understand, appraise, and communicate health information, while other experts perceived it as the interaction between individuals’ skills and abilities and the demands of the health system.

Respondents considered the following characteristics most important for a health literacy intervention model:

1. The model should take into account that individuals’ health literacy skills are influenced by their social context (e.g., family and peers).
2. The model should take into account that individuals’ health literacy skills may change during the life course (e.g., health education, cognitive decline).
3. The model should take into account that individuals’ health literacy skills are influenced by their personal characteristics.
4. The model should be flexible between different health contexts (e.g., differences in countries, types of care, etc.)
5. The model should be flexible in relation to various targets for interventions.

The modifiable determinants suggested by the respondents in the first round were split into four categories: (1) individual determinants (e.g., attitudes and skills to perform healthy behaviors or self-management), (2) contextual determinants (e.g., social cohesion, social support to enhance health behaviors), (3) determinants related to health professionals (e.g., skills to inform and educate people with low health literacy, skills to enable self-management), and (4) determinants related to the health system (e.g., cultural sensitivity, norms of professionals regarding health literacy). In the second round, respondents rated many of the modifiable determinants in all four categories as important for interventions on health literacy. Potentially modifiable determinants regarding professionals had the highest importance scores. Increasing awareness, knowledge, motivation, and skills of professionals were considered important aspects.

### 3.3. Consensus Meetings

Members of the IROHLA consortium present during the consensus meetings included staff members from universities, research institutes, international interest groups, non-governmental organizations, insurance companies, and ICT companies from nine countries in the European Union (a total of 21 organizations). Both consensus meetings were organized as parts of broader consortium meetings to discuss the progress of the IROHLA project.

In the consensus meetings, the nature of health literacy (i.e., skills and abilities of individuals vs. interaction between individuals’ skills and abilities and the demands of the health system) was discussed extensively. However, all participants agreed that the model should make clear that health literacy outcomes are the result of both individuals’ skills and abilities and the demands of the health system. Various participants also remarked that (1) the model should clearly show that health literacy affects health outcomes via intermediate outcomes, (2) that interventions to improve health literacy may also focus on individuals’ social contexts, and (3) that interventions may address both individuals with low health literacy and health service providers.

### 3.4. Development of a Comprehensive Health Literacy Intervention Model

**Figure 3:**
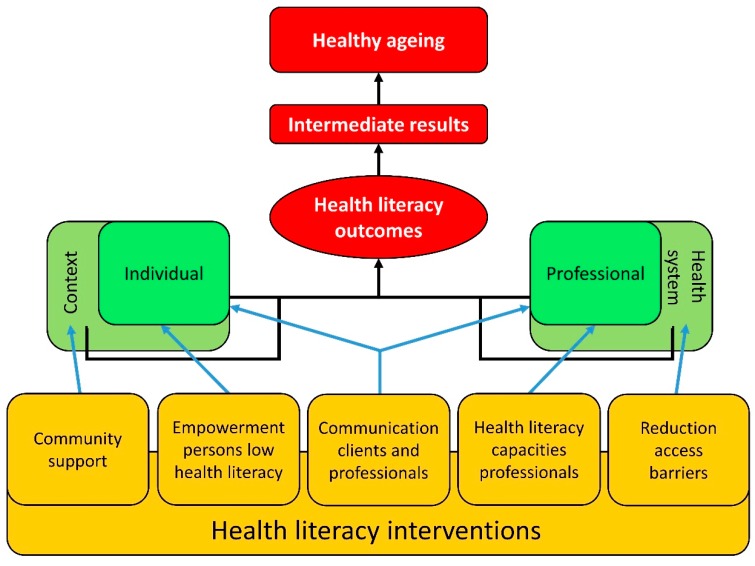
Final version of the health literacy intervention model.

Based on the results of our three research methods, we developed a final version of the health literacy intervention model (Figure 3). This final version focuses on the individual and the health professional as the main actors that together determine health literacy outcomes. In the model, both actors are viewed as part of a broader context. For the individual, this encompasses the social context (e.g., family members, friends, peers); and for the health professional, it constitutes the health system.

The model shows that better health literacy outcomes can be achieved when interventions target (combinations of) the following five factors:

1. The context of the individual, via interventions that strengthen the social support systems (e.g., family, peers, caregivers, communities).
2. The individual with low health literacy, via empowering interventions (e.g., person-centered capacity building and self-management).
3. The interaction between individual characteristics and the demands of the health system, via interventions to improve communication between individuals and health professionals.
4. Health professionals, via interventions aimed at improving their health literacy capacities (e.g., recognizing health literacy related problems, communication skills).
5. Improving communication and accessibility of health systems, via interventions aimed at reducing barriers to access or policies to improve quality of care or patient safety.

The relations in the model show the interaction between characteristics of the actors and their contexts, and how this eventually affects health literacy outcomes. These include knowledge, understanding information, self-efficacy, making informed decisions, self-management skills, social engagement and patient–provider trust. The term health literacy outcomes (rather than health literacy) was chosen to make the model suitable, regardless of whether health literacy is perceived as a characteristic of individuals, or a result of the interaction between individual skills and the demands of the healthcare context. The model also shows that health literacy outcomes affect healthy ageing via a series of potential intermediate outcomes (e.g., health behaviors, adherence, access to care).

Unlike the provisional intervention model, the final model does not distinguish between empowering activities and communication activities. These two types of activities were merged into one element in the model, as the distinction between the two is not clear-cut. Interventions may often contain elements of both types of activities, and affect both individuals or their context, and health professionals or the health system.

## 4. Discussion

Using a multimethod approach, we developed a comprehensive health literacy intervention model. Our model constitutes a valuable addition to the existing health literacy models because (1) it is among the few models that focus specifically on interventions to improve health literacy outcomes; (2) it is comprehensive and promotes synergistic interventions that involve multiple actors and their contexts; and (3) it views health literacy as an asset, rather than as a risk. These three characteristics of our model are described in more detail below.

As confirmed by the results of our literature review, our model is among the first to focus explicitly on interventions to improve health literacy outcomes. Most existing models focus mainly on the determinants and outcomes of health literacy. Many of these models have led to important insights into the potential determinants of health literacy and the mediators between health literacy and health outcomes, but they provide limited guidance for the development of comprehensive interventions to improve these outcomes. The most relevant suggestions concerning health literacy interventions described in other models [8,14,19,23,27] have been included in the development of our model. Its comprehensive focus on interventions is a major strength of the proposed model, as it encourages developers of interventions to address multiple factors simultaneously (i.e., skills of people with low health literacy, social support, capacities of health professionals, health systems).

We found only few existing health literacy models that focused explicitly on interventions in any way. This is probably because, until recently, health literacy research generally focused on defining the concept, developing measurement instruments, and identifying associations between health literacy and its determinants and outcomes. Only very recently has health literacy research shifted its focus towards the development and testing of interventions, and the available models do not yet reflect this new trend in the research. Our comprehensive health literacy intervention model fits with these recent developments, and it may therefore be used to further advance the field of health literacy research. By concretizing the necessary routes to be taken to improve health literacy outcomes, our model forms a bridge between existing health literacy models and actual practice.

Our comprehensive health literacy intervention model is supported by existing interventions on health literacy, and adequately captures the goals and targets of these interventions. In a recent study, the 15 most promising interventions aimed at improving the health literacy outcomes of older adults were selected and categorized by goals and target groups [30]. These interventions were found to address individuals at risk of having low health literacy, health professionals, or both these groups. Some interventions specifically addressed health professionals’ capacities to communicate effectively with people with low health literacy. Finally, various interventions focused on improving contextual support or facilitating the involvement of individuals with low health literacy in health systems. Other results of the IROHLA project also support the fit of the health literacy intervention model with the goals and targets of existing interventions. These results are described in a policy brief [31].

The main goal of our model is to conceptualize various ways to achieve better health outcomes for individuals with low health literacy. The model also provides a conceptual framework for dynamic interactions between individuals with low health literacy and service providers, in which the context plays a role on both sides. When an intervention consists of multiple activities (e.g., capacity building of professionals in combination with empowering of persons with low health literacy) that take place simultaneously and in alignment, the activities are likely to reinforce each other, making them more effective than when used in isolation. Interventions that focus on more than one factor related to health literacy have, indeed, been suggested to be more effective than interventions focusing on only a single factor [32]. Further research on such synergistic effects in health literacy interventions is needed to improve our knowledge of how to optimize the beneficial effects of such interventions.

At the basis of our model is the view of health literacy as an asset that needs to grow, rather than a risk factor that needs to be managed [20]. In this view, persons with low health literacy and health service providers can together become agents of change, in interventions calling for peer education and support. Together they can create a momentum by which new capacities lead to confidence in decision-making, leading in turn to the development of critical health literacy [29].

### 4.1. Strengths and Limitations

The main strength of our study was the use of multiple, complementary methods. Our literature review allowed us to build on existing knowledge regarding health literacy, while our use of online consultations and consensus meetings provided us with more practice-oriented knowledge.

Our study also had a number of limitations. First, we cannot rule out the possibility that we missed a small number of articles presenting a health literacy model in our literature review, as we only searched for articles in MEDLINE. However, to minimize the risk of missing relevant articles, we adopted a broad and systematic search strategy. We also used a systematic procedure to select articles, and we checked the references of selected articles for further possible sources, also if they were not available via MEDLINE. Second, although 68 health literacy experts took part in the online consultation, providing valuable, diverse, and overlapping input, we cannot assume full saturation. However, to compensate for this, we additionally employed consensus meetings with health literacy experts. We expect that this broad approach enhanced the wider applicability of our results and of our intervention model. Third, the online consultation procedure and the consensus meetings were conducted in the framework of the IROHLA project, which focused specifically on older adults (50+) in Europe. As the model is also supported by the results of the literature review, which focused on the general population of adults worldwide, we expect that the model is applicable for other adult populations. However, this deserves further study.

### 4.2. Implications for Practice and Research

Our comprehensive intervention model has a number of implications for practice. First, developers of interventions should consider adopting a comprehensive approach and using different intervention strategies to promote positive health literacy outcomes. Cross-sectoral and intersectoral collaborations may be necessary to accomplish this. Second, our model can be used in health education to make (future) health professionals more aware about health literacy and the importance of using a comprehensive approach. Third, our model emphasizes a dynamic concept of health literacy, where interactions between actors can modify outcomes of interventions, where “demand” and “supply” play a role and where one can expect changes over lifetime. The model can support the longitudinal provision of prevention, treatment, and care to people with low health literacy.

Further research is needed to strengthen the evidence base of our intervention model. First, additional intervention research is needed to assess which combinations of interventions create the most synergistic effects and improve health literacy outcomes most effectively. Second, as the effectiveness of health literacy interventions can be expected to be context-dependent [33], further research should determine which combinations of actions are most useful in which context. Third, longitudinal research is needed to understand changes in health literacy skills and factors contributing to such changes during the life course. Studies on these topics may lead to refinements of our intervention model and accelerate progress in health literacy intervention research.

## 5. Conclusions

Based on expert opinions and the existing literature, we developed a comprehensive health literacy intervention model. Our model can contribute to the improvement of health outcomes among people with low health literacy by supporting the development of more effective health literacy interventions.

## Supplementary Materials

The following are available online at http://www.mdpi.com/1660-4601/15/6/1268/s1, Table S1: Overview of health literacy models and frameworks.
