# Supplementary material for: Moving towards a Comprehensive Approach for Health Literacy Interventions: The Development of a Health Literacy Intervention Model
Source: Int J Environ Res Public Health. 2018 Jun 15;15(6):1268. doi: 10.3390/ijerph15061268 (PMC6025600; doi:10.3390/ijerph15061268)
Supplement: Supplementary file 1 [file ijerph-15-01268-s001.pdf]

Table S1. Overview of health literacy models and frameworks.

Source	Basis for model/framework	Description of the model/framework	Implications of the model/framework for interventions
Baker (2006) [1]	Model based on existing literature and expertise of the author.	The model focuses on health literacy in the general population. The model distinguishes between print literacy and oral literacy and includes both determinants and outcomes. It describes the personal capacities that determine individuals' levels of health literacy and the intermediate outcomes via which their health literacy influences health outcomes.	No specific implications of the model for interventions are discussed.
Dawkins-Moultin, McDonald, & McKyer (2016) [2]	Model based on the socioecological model (SEM) [3,4] and the principle of critical pedagogy [5,6].	The model focuses on health literacy in the general population and includes both determinants and outcomes. The model describes that health literacy is influenced by various levels of the health environment (interpersonal, community, societal). It also describes how agency mediates between health literacy and health outcomes and which factors moderate this relation.	The authors provide a general description of the implications of the model for interventions: "SEM indicates leverage points for interventions, while critical pedagogy clarifies the "how" or practical application."
Devraj & Gordon (2009) [7]	Model based on earlier models from the existing literature [1,8,9].	The model focuses on health literacy in patients with chronic kidney disease (CKD) and includes both determinants and outcomes. The model describes which individual factors and system factors determine patients' health literacy. It also includes various intermediate outcomes through which health literacy may affect health outcomes in CKD.	No specific implications of the model for interventions are discussed.
Harrington & Valerio (2014) [10]	Model based on results from a literature review, interviews with pediatric healthcare providers, and focus groups discussions with patients.	The model focuses on Verbal Exchange Health Literacy (VEHL) in the general population, which is considered the aspect of health literacy that concerns exclusively listening and speaking (as opposed to also including reading, writing, and numeracy). The model includes both determinants and outcomes. It lists which characteristics of patients, healthcare providers and the system, and their relations, affect VEHL. The model also describes the intermediate outcomes through which VEHL affects health outcomes.	No specific implications of the model for interventions are discussed.

Koh, Brach, Harris, & Parchman (2013) [11]	Model based on the expanded Care Model [12,13] and the Health Literacy Universal Precautions Toolkit [14].	The 'Health Literate Care Model' expands the Care Model by incorporating elements of health literacy. The Care Model focuses on healthcare organizations and can be used to make interactions between healthcare teams and patients as productive as possible. It consists of elements (e.g. decision support, community partners) that have effectively supported productive interactions at both the individual and the organizational level.	The authors provide a general description of the implications of the model for interventions: "The Health Literate Care Model represents a practical systems framework for organizations that aspire to adapt to all patients' health literacy challenges comprehensively, synergistically, and proactively."
Lee, Arozullah, & Cho (2004) [15]	Model based on existing literature.	The model focuses on the outcomes of health literacy in the general population. The model describes four intermediate outcomes through which health literacy affects health status, emergency care, and hospitalization. The intermediate outcomes are: (1) disease knowledge and self-care, (2) health risk behavior, (3) preventive care and routine physician visits, and (4) compliance with medications.	No specific implications of the model for interventions are discussed.
Mancuso (2011) [16]	Model based on a literature review.	The model focuses on health literacy in Indonesian asylum seekers in New Hampshire (USA). It distinguishes between fundamental literacy, scientific literacy, civic literacy, and cultural literacy. The model describes strategies that could be used in health education programs to overcome health literacy barriers.	A general description of the implications of the model for interventions is provided, supplemented with some specific suggestions: "A model to overcome health literacy for specific groups of individuals provides a framework for planning and subsequent action that guides the process of program development. Efforts to overcome health literacy must be bidirectional; attendant to community needs but also adding to provider knowledge and understanding."
McCormack, Thomas, Lewis, & Rudd (2017) [17]	Model based on the social ecological model (SEM) for social sciences [18].	The model focuses on health literacy and patient engagement in the general population. The model views health literacy and patient engagement as two integrated constructs. The model divides the environment in five ecological levels (individual, interpersonal, organizational, community, and macro policy). According to the model, all levels contain determinants that affect health literacy and patient engagement.	A general description of the implications of the model for interventions is provided and five interventions strategies are described: "The SEM emphasizes the importance of considering multiple levels of influence on individuals as a result of their physical and social environments" and "This paper describes five structured strategies for implementing interventions based on the 'health literacy SEM' and also provides ideas for how they can be combined for synergistic impact."

Nielsen-Bohlman, Panzer, & Kindig (2004) [19]	Framework based on existing concepts and definitions of health literacy.	The framework focuses on health literacy in general and includes both determinants and outcomes. The framework states that literacy is the foundation of health literacy, which itself forms the bridge between individual skills and abilities and the health context. Health literacy affects health outcomes and costs. The sectors that constitute the contexts of health literacy are culture and society, the health system, and the education system.	The authors give general suggestions for interventions: "These sectors [culture and society, the health system, and the education system] also provide intervention points that are both challenges and opportunities for improving health literacy."
Nutbeam (2000) [20]	Model based on existing health literacy research and earlier analyses of the determinants of health, and the definition of outcomes associated with health promotion activities [21].	The model focuses on health literacy in the health promotion context and includes both determinants and outcomes. The model views health literacy as a health promotion outcome that can be targeted by health promotion actions (e.g. health education) and affects health and social outcomes via intermediate outcomes (e.g. lifestyle).	The author describes how the model is relevant for interventions: "[The model] provides the bridge between an intervention and the goal of an intervention. Use of this model places health education and communication into the wider context of health promotion, and highlights health literacy as a key outcome from health education."
Nutbeam (2008) [22]	Models based on various earlier studies and models [1,9,23,24].	Two models presented, both of which focus on health literacy in the general population. The models compare two approaches to health literacy: (1) health literacy as a risk factor that needs to be managed and (2) health literacy as an asset to be built. Both models describe how health literacy affects health outcomes via intermediate outcomes. The model that approaches health literacy as an asset also describes determinants of health literacy.	No specific implications of the model for interventions are discussed.
Paasche-Orlow & Wolf (2007) [9]	Model based on analyses of pre-existing findings in medical and public health literature on health literacy.	The model focuses on health literacy in the general population and includes both determinants and outcomes. The model describes which personal and contextual factors determine an individual's level of health literacy and lists three intermediate outcomes (access and utilization of healthcare, patient-provider interaction, and self-care) through which health literacy affects health outcomes.	No specific implications of the model for interventions are discussed.

Rootman & Ronson (2005) [25]	Framework loosely based on the work carried out for the Ontario Public Health Association and Frontier College project [26]. Revisions based on feedback received in focus group discussions.	The model focuses on health literacy in the general population in Canada. The model includes both determinants and outcomes. It lists a number of actions and individual and contextual determinants that determine people's general literacy, health literacy, and other forms of literacy. The model also describes both direct and indirect routes (i.e. via intermediate outcomes) through which health literacy affects health status and quality of life.	The authors provide specific suggestions for targets for interventions: "As suggested by the conceptual framework, interventions addressing literacy and health concerns include health communication, education/training (capacity development), community and organizational development, and policy development."
Roter, Erby, Larson, & Ellington (2007) [27]	Based on the results of an earlier study conducted by the authors [28].	The model focuses on health literacy demands in genetic counseling dialogue and includes both determinants and outcomes. The model describes pathways through which language characteristics might influence client-provider interaction. The model also lists a number of cognitive, psychological, behavioral, health, societal and provider outcomes that are potentially affected by the quality of client-provider interaction.	No specific implications of the model for interventions are discussed.
Shreffler-Grant, Nichols, Weinert, & Ide (2013) [29]	Model based on a comprehensive review of the literature and a review of data from the authors' prior research [30-32].	The model focuses on complementary and alternative medicine (CAM) health literacy in the United States. The model includes both determinants and outcomes. It describes how environmental and individual antecedents affect CAM health literacy, which in turn affects informed self-management of health.	The authors provide a general description of the implications of the model for interventions: "The constructs and concepts within [the model] can be used to identify points of intervention for research or for clinical practice."
Sørensen et al. (2012) [33]	Model based on a systematic review of the literature on existing models and definitions of health literacy.	The model focuses on health literacy in the general population and includes both determinants and outcomes. The model mainly focuses on the concept of health literacy itself and describes the main dimensions of health literacy (i.e. accessing, understanding, appraising, and applying health information) and the role of health literacy in healthcare, disease prevention, and health promotion. The model also describes the main consequences of health literacy.	The authors provide a general description of the implications of the model for interventions: "[The model] can support the practice of healthcare, disease prevention and health promotion by serving as a conceptual basis to develop health literacy enhancing interventions."

Squiers, Peinado, Berkman, Boudewyns, & McCormack (2012) [34]	Model based on a review of existing theoretical frameworks on health literacy.	The model focuses on general health literacy skills (i.e. print literacy, communication, information seeking, and eHealth) and includes both determinants and outcomes. The model describes the full pathway from determinants, via health literacy skills, via comprehension of stimuli, and via mediators to health-related behaviors and outcomes. The influences of health literacy demands and the ecological context on this pathway are also acknowledged.	The authors provide a general description of the implications of the model for interventions: "The conceptual framework can be used to guide the development of interventions to improve the health literacy of individuals. The framework identifies barriers to acquiring health literacy skills."
Vellar, Mastroianni, & Lambert (2017) [35]	Framework based on a literature review, a consultation process, and a pilot of health literacy strategies. The framework is underpinned by the principle of 'universal precautions' [36] and incorporates elements from other frameworks [37,38].	The framework focuses on health literacy in health systems and views health literacy as both an outcome and a process. The framework was specifically designed for implementation in the Illawarra Shoalhaven Local Health District in Australia. The framework describes five key goals (e.g. ensuring effective communication, integrating health literacy into education) that health systems should reach in order to ensure that consumers can effectively access, understand, and use health information and services.	The authors describe the results of the implementation of the framework: "Implementation of the framework has resulted in tangible improvements at several levels. The development of [the framework] was an important first step to embedding health literacy into health systems. The framework was designed as a systems approach."
Von Wagner, Steptoe, Wolf, & Wardle (2009) [39]	Framework based on established constructs from social cognition models of health.	The model focuses on health literacy in the general population and includes both determinants and outcomes. The model describes the routes through which health literacy and its determinants affect health actions via intermediate outcomes. The intermediate outcomes in the framework consist of sociocognitive and psychological determinants in the motivational and volitional phase.	The authors describe the value of the framework for interventions and provide examples of relevant interventions: "The framework can be used to design interventions in each of the three domains of health actions [access and use of health services, patient–provider interactions, and management of health and illness]."
Yin, Jay, Maness, Zabar, & Kalet (2015) [40]	Framework based on the concept of Educationally Sensitive Patient Outcomes (ESPOs).	The model focuses on physician education and views health literacy as an intermediate outcome between physician education and patient health. The model describes how physician education leads to the ability in physicians to address patient health literacy. Via patient knowledge and skills and patient engagement, this eventually leads to maximized patient health.	The authors provide a general description of the value of the model for interventions: "The ESPO framework allows us to identify curricular interventions likely to lead to improvement in patient capacities [i.e. health literacy] directly related to important health outcomes."

Yip (2012) [41]	Model based on an extensive literature review.	The model focuses on health literacy in populations with limited English proficiency in the context of communication platforms. The model includes both determinants and outcomes. The model distinguishes between five core health literacy skills (listening, speaking, reading, writing, and numeracy). The linear model describes the route from health literacy and its sources to health outcomes. Intermediate outcomes are communication platforms, health decisions and health-related activities.	The authors provide suggestions for interventions, based on the model: "The new focus of health literacy assessment should be on identifying individual strengths and weaknesses in each of the core skills. Health professionals can then leverage their patients' existing health literacy skills to promote engagement in care delivery. Health professionals can expand their patients' communication capacities by designing interventions that enhance the skills they need."
Yuen et al. (2015) [42]	Model developed using a concept mapping approach. Concept mapping workshops were held with people with cancer and their caregivers.	The model focuses on health literacy in caregivers of people with cancer. This circular conceptual model reflects six themes and seventeen subthemes related to cancer caregiver health literacy. The main themes are access to information, understanding information, relationship with healthcare providers, relationship with care recipients, support systems, and managing challenges of caregiving.	The authors provide a general description of the implications of the model for interventions: "The [concepts within the model] provide the first insights into targeted areas for the development of new measures of cancer caregiver health literacy. A measure that accurately assesses caregiver health literacy has the potential to identify key intervention."

References

1. Baker, D.W. The meaning and the measure of health literacy. *J. Gen. Intern. Med.* **2006**, *21*, 878-883, doi: 10.1111/j.1525-1497.2006.00540.x.
2. Dawkins-Moultin, L.; McDonald, A.; McKyer, L. Integrating the principles of socioecology and critical pedagogy for health promotion health literacy interventions. *J. Health Commun.* **2016**, *21*, 30-35, doi: 10.1080/10810730.2016.1196273.
3. McLeroy, K.R.; Bibeau, D.; Steckler, A.; Glanz, K. An ecological perspective on health promotion programs. *Health Educ. Q.* **1988**, *15*, 351-377, doi: 10.1177/109019818801500401.
4. Stokols, D. Translating social ecological theory into guidelines for community health promotion. *Am. J. Health Promot.* **1996**, *10*, 282-298, doi: 10.4278/0890-1171-10.4.282.
5. Wallerstein, N.; Bernstein, E. Empowerment education: Freire's ideas adapted to health education. *Health Educ. Q.* **1988**, *15*, 379-394, doi: 10.1177/109019818801500402.
6. Rhem, J. Critical Pedagogy. *NTLF* **2013**, *22*, 1-12, doi: 10.1002/ntlf.20011.
7. Devraj, R.; Gordon, E.J. Health literacy and kidney disease: Toward a new line of research. *Am. J. Kidney Dis.* **2009**, *53*, 884-889, doi: 10.1053/j.ajkd.2008.12.028.
8. Pignone, M.P.; DeWalt, D.A. Literacy and health outcomes: Is adherence the missing link? *J. Gen. Intern. Med.* **2006**, *21*, 896-897, doi: 10.1111/j.1525-1497.2006.00545.x.
9. Paasche-Orlow, M.K.; Wolf, M.S. The causal pathways linking health literacy to health outcomes. *Am. J. Health Behav.* **2007**, *31 Suppl 1*, S19-S26, doi: 10.5555/ajhb.2007.31.supp.S19.
10. Harrington, K.F.; Valerio, M.A. A conceptual model of verbal exchange health literacy. *Patient Educ. Couns.* **2014**, *94*, 403-410, doi: 10.1016/j.pec.2013.10.024.
11. Koh, H.K.; Brach, C.; Harris, L.M.; Parchman, M.L. A proposed 'health literate care model' would constitute a systems approach to improving patients' engagement in care. *Health Aff.* **2013**, *32*, 357-367, doi: 10.1377/hlthaff.2012.1205.
12. Wagner, E.H.; Austin, B.T.; Von Korff, M. Organizing care for patients with chronic illness. *Milbank Q.* **1996**, *74*, 511-544, doi: 10.2307/3350391.
13. Barr, V.J.; Robinson, S.; Marin-Link, B.; Underhill, L.; Dotts, A.; Ravensdale, D.; Salivaras, S. The expanded Chronic Care Model: An integration of concepts and strategies from population health promotion and the Chronic Care Model. *Hosp. Q.* **2003**, *7*, 73-82, doi: 10.12927/hcq.2003.16763.
14. DeWalt, D.A.; Broucksou, K.A.; Hawk, V.; Brach, C.; Hink, A.; Rudd, R.; Callahan, L. Developing and testing the health literacy universal precautions toolkit. *Nurs. Outlook* **2011**, *59*, 85-94, doi: 10.1016/j.outlook.2010.12.002.
15. Lee, S.D.; Arozullah, A.M.; Cho, Y.I. Health literacy, social support, and health: A research agenda. *Soc. Sci. Med.* **2004**, *58*, 1309-1321, doi: 10.1016/S0277-9536(03)00329-0.
16. Mancuso, L. Overcoming health literacy barriers: A model for action. *J. Cult. Divers.* **2011**, *18*, 60-65.
17. McCormack, L.; Thomas, V.; Lewis, M.A.; Rudd, R. Improving low health literacy and patient engagement: A social ecological approach. *Patient Educ. Couns.* **2017**, *100*, 8-13, doi: 10.1016/j.pec.2016.07.007.
18. Bronfenbrenner, U. Toward an experimental ecology of human development. *Am. Psychol.* **1977**, *32*, 513-531, doi: 10.1037/0003-066X.32.7.513.

19. Nielsen-Bohlman, L.; Panzer, A.M.; Kindig, D.A. *Health literacy: A prescription to end confusion*; National Academies Press: Washington, DC, USA, 2004.
20. Nutbeam, D. Health literacy as a public health goal: A challenge for contemporary health education and communication strategies into the 21st century. *Health Promot. Internation.* **2000**, *15*, 259-267, doi: 10.1093/heapro/15.3.259.
21. Nutbeam, D. Health outcomes and health promotion: Defining success in health promotion. *Health Promot. J. Aust.* **1996**, *6*, 58-60.
22. Nutbeam, D. The evolving concept of health literacy. *Soc. Sci. Med.* **2008**, *67*, 2072-2078, doi: 10.1016/j.socscimed.2008.09.050.
23. Imel, S. *Using adult learning principles in adult basic and literacy education*; Centre for Education and Training for Employment: Columbus, OH, USA, 1998. Available online: https://files.eric.ed.gov/fulltext/ED425336.pdf (accessed on 12 February 2018).
24. Freebody, P.; Luke, A. 'Literacies' programs: Debates and demands in cultural context. *Prospect: An Australian Journal of TESOL* **1990**, *5*, 7-16.
25. Rootman, I.; Ronson, B. Literacy and health research in Canada: where have we been and where should we go? *Can. J. Public Health* **2005**, *96 Suppl 2*, S62-S77.
26. Perrin, B. *Literacy and health – Making the connection: The research report of the Literacy and Health Project, Phase one: Making the world healthier and safer for people who can't read*; Ontario Public Health Association and Frontier College: Toronto, Ontario, Canada, 1990. Available online: http://www.opha.on.ca/resources/literacy1research.pdf (accessed 12 February 2018).
27. Roter, D.L.; Erby, L.H.; Larson, S.; Ellington, L. Assessing oral literacy demand in genetic counseling dialogue: Preliminary test of a conceptual framework. *Soc. Sci. Med.* **2007**, *65*, 1442-1457, doi: 10.1016/j.socscimed.2007.05.033.
28. Roter, D.; Ellington, L.; Erby, L.H.; Larson, S.; Dudley, W. The Genetic Counseling Video Project (GCVP): Models of practice. *Am. J. Med. Genet. C. Semin. Med. Genet.* **2006**, *142C*, 209-220, doi: 10.1002/ajmg.c.30094.
29. Shreffler-Grant, J.; Nichols, E.; Weinert, C.; Ide, B. The Montana State University conceptual model of complementary and alternative medicine health literacy. *J. Health Commun.* **2013**, *18*, 1193-1200, doi: 10.1080/10810730.2013.778365.
30. Nichols, E.; Sullivan, T.; Ide, B.; Shreffler-Grant, J.; Weinert, C. Health care choices: Complementary therapy, chronic illness, and older rural dwellers. *J. Holist. Nurs.* **2005**, *23*, 381-394, doi: 10.1177/0898010105281088.
31. Shreffler-Grant, J.; Weinert, C.; Nichols, E.; Ide, B. Complementary therapy use among older rural adults. *Public Health Nurs.* **2005**, *22*, 323-331, doi: 10.1111/j.0737-1209.2005.220407.x.
32. Shreffler-Grant, J.; Hill, W.; Weinert, C.; Nichols, E.; Ide, B. Complementary therapy and older rural women: Who uses it and who does not? *Nurs. Res.* **2007**, *56*, 28-33.
33. Sørensen, K.; Van den Broucke, S.; Fullam, J.; Doyle, G.; Pelikan, J.; Slonska, Z.; Brand, H. Health literacy and public health: A systematic review and integration of definitions and models. *BMC Public Health* **2012**, *12*, 80, doi: 10.1186/1471-2458-12-80.
34. Squiers, L.; Peinado, S.; Berkman, N.; Boudewyns, V.; McCormack, L. The health literacy skills framework. *J. Health Commun.* **2012**, *17 Suppl 3*, 30-54, doi: 10.1080/10810730.2012.713442.
35. Vellar, L.; Mastroianni, F.; Lambert, K. Embedding health literacy into health systems: A case study of a regional health service. *Aust. Health Rev.* **2017**, *41*, 621-625, doi: 10.1071/AH16109.
36. Walsh, C.; Shuker, C.; Merry, A.F. Health literacy: From the patient to the professional to the system. *N. Z. Med. J.* **2015**, *128*, 10-16.

37. de Savigny, D.; Adam, T. *Systems thinking for health systems strengthening*; Alliance for Health Policy and Systems Research, World Health Organization: Geneva, Switzerland, 2009.
38. Brach, C.; Keller, D.; Hernandez, L.M.; Baur, C.; Parker, R.; Dreyer, B.; Schyve, P.; Lemerise, A.J.; Schillinger, D. *Ten attributes of health literate health care organizations*; Institute of Medicine: Washington, DC, USA, 2012.
39. Von Wagner, C.; Steptoe, A.; Wolf, M.S.; Wardle, J. Health literacy and health actions: A review and a framework from health psychology. *Health Educ. Behav.* **2009**, *36*, 860-877, doi: 10.1177/1090198108322819.
40. Yin, H.S.; Jay, M.; Maness, L.; Zabar, S.; Kalet, A. Health Literacy: An Educationally Sensitive Patient Outcome. *J. Gen. Intern. Med.* **2015**, *30*, 1363-1368, doi: 10.1007/s11606-015-3329-z.
41. Yip, M. A health literacy model for limited English speaking populations: Sources, context, process, and outcomes. *Contemp. Nurse* **2012**, *40*, 160-168, doi: 10.5172/conu.2012.40.2.160.
42. Yuen, E.Y.N.; Dodson, S.; Batterham, R.W.; Knight, T.; Chirgwin, J.; Livingston, P.M. Development of a conceptual model of cancer caregiver health literacy. *Eur. J. Cancer Care* **2016**, *25*, 294-306, doi: 10.1111/ecc.12284.
